# Biodegradable Nanocomposite Microcapsules for Controlled Release of Urea

**DOI:** 10.3390/polym13050722

**Published:** 2021-02-26

**Authors:** Jessica de Carvalho Arjona, Maria das Graças Silva-Valenzuela, Shu-Hui Wang, Francisco Rolando Valenzuela-Diaz

**Affiliations:** Department of Metallurgical and Materials Engineering, Polytechnic School, University of Sao Paulo, Av. Prof. Mello Moraes 2463, Sao Paulo 05508-030, Brazil; gracavalenzuela@usp.br (M.d.G.S.-V.); wangshui@usp.br (S.-H.W.); frrvdiaz@usp.br (F.R.V.-D.)

**Keywords:** PHB, urea, microcapsules, encapsulation, biodegradation

## Abstract

Urea is the most used fertilizer around the world as the main source of nitrogen to soil and plants. However, the administration of nitrogen dosage is critical, as its excess can be harmful to the environment. Therefore, the encapsulation of urea to achieve control on its release rates has been considered in several areas. In this work, encapsulation of urea by biodegradable polymer poly(3-hydroxybutyrate) (PHB) and its nanocomposites, namely PHB/MMT and PHB/OMMT, producing microcapsules by emulsion method is carried out. MMT and OMMT refer to Brazilian clays in a natural state and organophilized, respectively. In addition, the microcapsules are thus prepared to have their physicochemical characteristics investigated, then tested for biodegradation. Increment of microcapsules’ crystallinity due to the increased amount of poly(vinylacetate) (PVA), as emulsifier agent in the continuous phase, was confirmed by X-ray diffractometry (XRD) and atomic force microscopy (AFM). The presence of urea within microcapsules was verified by XRD, thermogravimetric analysis (TGA) and scanning electron microscopy (SEM). The soil biodegradation assessments showed that PHB/OMMT microcapsules present higher degradation rates in sandy soils. The overall results suggest that the composites performed better than neat PHB and are very promising; moreover, PHB/OMMT microcapsules proved to be the best candidate for the controlled-release of urea in soils.

## 1. Introduction

The growth of agricultural production has raised in the last few decades due to the high demand and continuous introduction of updated technology and management of crops. In consequence, it is necessary to use more agrochemical products, which will control the conditions of plantations and enable it. However, the indiscriminate use of those substances can cause environmental problems, physical and chemical soil changes, water contamination, and harm to human health [1,2,3].

Fertilizers with nitrogen (N) are the most important because this element is one of the great soil nutrients as well as essential to innumerous plantations. Urea is the most used fertilizer in agriculture to provide that nutrient to plants. Nevertheless, this substance can react with water, turning into nitrates, or even decomposing into ammonia, which is a toxic gas released to the environment before reaching the plants [1,4].

Controlled delivery systems have been studied to maximize efficiency and reduce the necessary quantity of fertilizers and pesticides, therefore, increasing its safety and efficiency while guaranteeing a most effective way to preserve soil fertility, improve agriculture production and food safety [1]. The most common release system studied is the coating of urea with wax, polymers, or sulfur. These coatings help reduce the dissolution in water and, in consequence, limit the eutrophication of rivers [2,5].

Da Costa et al. (2019) [4] studied urea granules coating through a suspension of biodegradables polymers, glycerol, distilled water, and talc. This work aimed to evaluate the nitrogen loss rate by volatilization, which is the principal in-field urea deprivation and is associated with urea hydrolysis and depends on the physical-chemical soil characteristics. Natural polymers have also been suggested as good candidates to coat urea granules, decreasing the release rate of urea and potential damages to the environment. Up to 50% reduction of N loss by volatilization route compared to uncoated granules was reported, and the coating layer presented more effectiveness in accordance with its homogeneity and diminished porosity. Similar studies were developed by Qiao et al. (2016) and Yu Li (2019) [6,7], reducing the rate of urea release by coating with biopolymers and paraffin.

Many release systems of fertilizers use as transport vector inorganic materials such as clays [8], zeolites, double layer hydroxides [5], and some oxides [9]. Although some studies presented a reduction of loss of these substances to the soil, air, and water, they have not presented satisfactory results about a decrease of lixiviation, which accelerates the release of fertilizers to the environment [2].

The microencapsulation of fertilizers is a method that has been considered for its controlled-release. Araújo et al. (2017) [1] dispersed urea, peat, and humic acids in chitosan and obtained controlled-release microspheres by emulsion methods. The authors studied the interaction of those components and their influence on the release process. Urea release was analyzed in an aqueous environment, at different pH and in soil. They observed that in acid pH, urea was more easily released than in basic one, crashes in shells’ surface and that the presence of peat and humic acids increased the shell resistance and the controlling of release rate, with linear curve and lower release rate. Similar studies using microparticles of chitosan for controlled-release of fertilizers were developed by Santos et al. (2015) and França et al. (2018) [10,11], showing the increase of adsorption and more control in the release rate.

PHB is a linear polyester with a semi-crystalline structure [12] and mechanical properties similar to polypropylene and polystyrene [13,14]. However, PHB presents limited applicability because it is brittle, has low stability in thermal processing, and has low processability in comparison to polymers made of fossil fuel [14,15]. To improve its processability and thermal properties while keeping its mechanical strength, formulating its nanocomposites has been suggested [14], including those prepared with nanoclay [16,17]. The preparation of nanocomposites is easily carried out using ordinary polymer melting processes [2,14,17] and through dissolution, dispersion, and emulsion in a fluid medium followed by removing the liquid completely and recover the solid polymer [18,19].

PHB is a biopolymer widely studied and used when biodegradation enforcement is required since there are numerous depolymerase enzymes capable of decomposing PHB as well as different microorganisms, as bacteria and fungi, synthesize it. Thus, PHB can be degraded in numerous environments like soil, aqueous and saline medium, and even in the human body, and no harmful substance comes from its metabolization [12,18,20]. PHB microcapsules have a wide application due to the versatility of PHB as a vehicle to release drugs [21,22,23], pesticides, fertilizers [2], and proteins [24].

Polymeric nanocomposites having clay as the nanometer particles in the PHB matrix are important materials with improved properties, such as mechanical, thermal, barrier and biodegradability. In contrast, nanocomposite microcapsules contribute with easy handling, good surface structure, higher biodegradability rate, and better protection to active chemicals [19,25,26].

Several methods are used to prepare microcapsules having polymer matrices, such as emulsion, spray-drying, freeze-drying, coaxial electrospray systems, coacervation, in situ polymerization, extrusion, supercritical fluid technology, and fluidized-bed-coating. The emulsion method is widely used to encapsulate bioactive substances in pharmaceutical and food areas, and the final product can be preserved in water or be dried and used as a powder. This method depends on whether the encapsulated chemical is hydrophilic or hydrophobic; in the case of hydrophilic substances, as urea, water–oil–water (*w*/*o*/*w*) emulsion is used [27,28].

The preparation of microcapsules by emulsion method requires a sequential mixture of a water/oil emulsion of the polymer, a solution containing a dispersing agent, and an aqueous solution of the active substance to be encapsulated. Furthermore, to obtain the droplets, a so-called continuous phase made of water and dispersing agent is necessary, as it is where the polymer *w/o* emulsion is dropped under stirring to jellify the polymer. Therefore, there are at least two different liquid systems to be analyzed that can impact the microcapsule properties, the water/oil emulsion and the continuous phase. This method can influence PHB microcapsules’ characteristics as crystallinity, size, and shape. The crystallinity is associated with the roughness of microcapsules, and it is related to solvent extraction and affects the porosity on the microcapsule surface [29,30,31].

The goal of this work is to prepare urea encapsulated by PHB and PHB nanocomposite microcapsules through the emulsion method and investigate the influence of the preparation method on the microcapsules’ characteristics and their biodegradability. Finally, the potential use of the microcapsules as controlled-release systems aiming to avoid the ecological problems caused by excessive urea release in the environment is certainly a primary objective.

## 2. Materials and Methods

### 2.1. Materials

A Brazilian Northeast smectite from Vitoria da Conquista, Bahia, Brazil, was used; it is a polycationic clay received in its natural state. Poly(3-hydroxybutyrate) (PHB) (M_w_ = 600,000 g/mol circa) was gently supplied by PHB Industrial S/A (Serrana, Brazil). The commercial ammonium quaternary salts Sunquart CT 50 in ethanol solution (50%) (C_19_H_44_NCl) (molar mass of 321.5 g/mol) were supplied by Polytechno (Guarulhos, Brazil). PVA, urea, chloroform, and sodium carbonate (99.5%) were supplied by Sigma-Aldrich (Milwaukee, WI, USA). The compatibilizer is a Manioc Starch, Tapioca Pure (TP), molar mass similar to potato starch, was gently supplied by AkzoNobel (Itupeva, Brazil).

### 2.2. Methods

#### 2.2.1. Clay Purification

The smectite utilized in the present work was purified before use. First, a sample of the original clay was stirred in water (15% *w/w*) at 14,000 rpm for 20 min to form an aqueous dispersion and left to rest. After resting, the dispersion was spread in three phases with different colors, which were carefully separated. The intermediate phase, a red clay, here denominated MMT, was used in this work [32].

#### 2.2.2. Clay Organofilization

An MMT/water dispersion (4% *w/w*) was prepared by stirring the mixture at 14,000 rpm for 20 min. Then, Na_2_CO_3_ in the proportion of 1 mol/kg (of clay) was added to perform cation exchange by mixing at 1500 rpm for 20 min., followed by dropwise addition of ammonium quaternary salt solution in the proportion of 1 mol/kg under 1500 rpm stirring for 30 min. The mixture was left to rest overnight; afterward, the supernatant was removed from the dispersion, and the resultant OMMT was thoroughly washed with distilled water and filtered. The resultant OMMT was dried at 60 °C for 24 h and sieved through a 200-mesh sieve.

#### 2.2.3. Preparation of Nanocomposite Dispersions

Nanocomposites were obtained by the dispersion method. Chloroform was used as the solvent to prepare two kinds of 0.4% (*w/w*) clay dispersions, one of OMMT and the other of MMT, and a 3.6% (*w/w*) PHB solution. Clay dispersions were prepared using magnetic stirring for 3 h at room temperature, while PHB solution was kept under magnetic stirring for 30 min at 40 °C. The composite dispersion was prepared by dropwise addition of the PHB solution into each clay dispersion under magnetic stirring. Through this method, three different polymer systems were used to prepare the microcapsules, namely PHB, a homopolymer, PHB/MMT and PHB/OMMT, two nanocomposites. To prepare PHB/OMMT nanocomposite, TP was added in OMMT dispersion in a proportion of 1:2 of TP/OMMT.

#### 2.2.4. Preparation of Microcapsules

Microcapsules were obtained by the emulsion-diffusion method. First, an emulsion w/o was prepared stirring aqueous urea solution, organic solution (PHB or PHB/MMT or PHB/OMMT in chloroform), and PVA aqueous solution by mechanic stirring at 150 rpm, for 2 min. This emulsion was dropwise added to a PVA solution (continuous phase) at 1500 rpm mechanical stirring, which was maintained under stirring for another 2 h, after the addition of the emulsion, to promote chloroform evaporation. When the stirring is stopped, microcapsules were obtained settled at the bottom of the beaker. To evaluate the influence of the PVA concentration in the continuous phase in the microcapsule’s formation, they were also prepared with PVA at two different concentrations, 0.5% and 2.0%. The stable *w/o* emulsion (first emulsion) presents PVA at 1.0%.

### 2.3. Characterizations

#### 2.3.1. X-ray Diffractometry (XRD)

XRD spectra were obtained with a Rigaku MiniFlex600 X-ray diffractometer system with Cu K_α_ radiation (Tokyo, Japan). Scans were recorded in the range of 2Ɵ = 2°–30° at 2°/min with an X-ray tube operated at 30 kV and 10 mA. The XRD curves were used to verify the crystallinity of microcapsules performing the deconvolution of peaks using a Gaussian distribution (R^2^ = 0.999).

#### 2.3.2. ATR-FTIR Spectroscopy (FTIR)

FTIR spectra were obtained with a Thermo Fisher Scientific Nicolet Is5 using ATR mode and ZnSe crystal (Madison, WI, US).The spectra were collected after 16 scans, in the range 4000 cm^−1^ to 600 cm^−1^, 2 cm^−1^ of the resolution, and air spectrum were collected as the background just before analyzing the sample.

#### 2.3.3. Scanning Electron Microscopy (SEM)

Microcapsules morphology and sizes were observed using SEM, Cambridge Stereoscan 4040 by FEI Company (Eindhoven, The Netherlands) equipped with secondary electron and backscattered electron detectors. The microcapsule samples were metalized with gold prior to analysis.

#### 2.3.4. Atomic Force Microscopy (AFM)

Samples of PHB microcapsules made with 0.5% and 2.0% PVA were analyzed by AFM in the tapping mode using Bruker equipment (Santa Barbara, CA, USA). Scanner “E” was used, scan sizes of 6 μm with 512 points/line. The equipment software, NanoScope Analysis 1.5 by Bruker (Santa Barbara, CA, USA), was used in the measurements of mean roughness (r_a_) and mean quadratic roughness (r_q_), all performed in triplicate. To avoid the influence of microcapsules curvature, the AFM images were treated with the “Flatten” filter available in the NanoScope Analysis software.

#### 2.3.5. Thermogravimetric Analysis (TGA)

TGA was carried out in Argon atmosphere, at a rate of 10 K/min using a thermogravimetric analyzer STA 449F1 Jupiter^®^ by NETZSCH (Selb, Germany), in the temperature range of 30 to 900 °C for the clays’ samples and in the range of 30 to 600 °C for the polymeric films.

#### 2.3.6. Biodegradation Test (ASTM 5988-92)

Biodegradation test of microcapsules was carried out according to ASTM 5988–92 “Standard test method for determining aerobic biodegradation of plastic materials in soil”. A natural soil from the University of Sao Paulo (USP) with active microbiota was used without drying or freezing process. The soil was investigated in its maximum water holding capacity (ASTM D425), with the appropriate number of ashes and organic material, soil’s pH (ASTM D4972), and soil texture [33]. All analyses were carried out in triplicate.

## 3. Results

### 3.1. Clays

**Figure 1:**
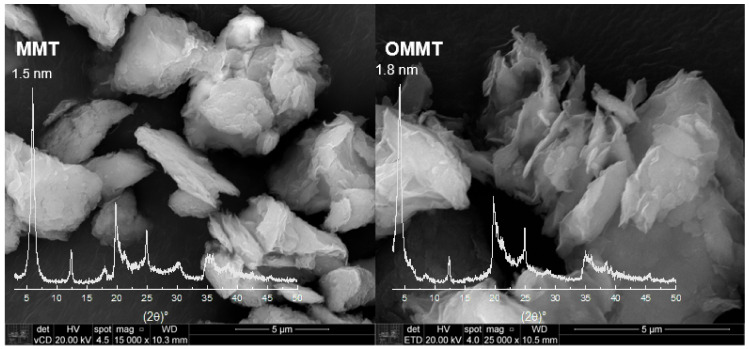
XDR and scanning electron microscopy of montmorillonite (MMT) and organophilized montmorillonite (OMMT) samples (bar indicates 5 μm).

Figure 1 shows the XRD patterns of MMT and OMMT samples, which present reflection due to an interplanar spacing d001– 1.5 nm and another d001– 0.7 nm, which are related to clay minerals montmorillonite (MMT) [34] and kaolin (K), respectively, in the proportion of 62.3% and 37.7%, respectively, as shown previously [32].

The OMMT and MMT present similar XRD spectra, although it is possible to observe that there is a shift of the basal peak, and the lamellar distance increased to 1.8 nm for OMMT compared to MMT. This rise is due to the intercalation of the quaternary salt, indicating that the clay organophilization was successful. Moreover, it can be used to verify the conformation of the organic salt in the inner layers of the clay, and in this case, the preferential conformation is in a bilayer arrangement [35]. SEM micrographs for OMMT and MMT samples are shown in Figure 1. It is possible to observe that after the organophilization process, the clay particles showed themselves more exfoliated.

TGA curve of MMT (Figure S1, Supplementary Material) presents the first step of weight loss (stage I, 13.6% of weight) up to approximately 200 °C, due to water evaporation, molecules adsorbed on the clay [34,36]. In stage II, the loss rate occurs until around 500 °C, when starts the stage III up to 770 °C with 6.4% of weight loss due to the clay dihydroxylation [37], and finally appears the stage IV, where there is no more reduction of weight and the residue, 82%, remains up to 900 °C. On the other hand, the OMMT curve presents a first weight loss (stage I, starts around 100 °C) of only 2.4% due to the water evaporation. This result was expected since the organoclay presents a hydrophobic character and has on its surface fewer water molecules than the natural clay. The stages II and III spread from around 200 to 770 °C; in this range, the decomposition of the organic salt and the dihydroxylation of clay occur, and the mass loss sums 27.3%. As both clays, OMMT and MMT, have the same constitution of clay minerals, they have the same proportion of dihydroxylation, 6.4%, hence, the weight decreasing due to the decomposition of quaternary salt was 20.9%.

FTIR spectra of OMMT and MMT (Table S1, Supplementary Material) showed bands around 815 cm^−1^ and 1026 cm^−1^, typically found in bentonite clays. The former is related to dioctahedral clays, and the latter is associated with the Si–O bonds stretching [34]. The bands around 1600 cm^−1^ and 3500 cm^−1^ are related to the vibration of O–H bonds of water molecules; around 690 cm^−1^ is related to the vibration bonds of Si–O-Mg, and the band around 1108 cm^−1^ is due to the stretching band of Si–O bonds [34,37]. The bands around 3659 cm^−1^ are due to the stretching vibration of OH bonds on the surface and inner part of the kaolin particles [32,38]. OMMT also showed two more bands around 2931 cm^−1^ and 2838 cm^−1^; the latter is related to the scissor vibrations of C–H_3_ bonds and the former to stretching of C–H bonds. Furthermore, around 1400 cm^−1^, there is an increase in the number of bands, and those are related to the CH_2_ bonds [39].

### 3.2. Microcapsules’ Crystallinity

To verify the influence of the concentration of PVA in the continuous phase in the PHB microcapsules formation, two types of them were prepared: one with 0.5% PVA and another with 2.0%. It is possible to observe the morphology of the surface of those particles and qualitatively compare their roughness by SEM (Figure S2a,b, Supplementary Material), as the microcapsules from 2.0% PVA present a larger number of assorted-sized holes and irregularities compared to that from 0.5% PVA. In addition, AFM tapping mode was used to determine roughness (Figure S2c,d, Supplementary Material), and microcapsules made with 0.5% of PVA displays r_a_ (mean roughness) of 78.4 nm and r_q_ (mean quadratic roughness) of 95.9 nm, while those obtained with 2.0%, r_a_ of 126.6 nm and r_q_ of 154.7 nm. These results indicate that a high concentration of emulsifier agents increases microcapsule roughness.

The microcapsules’ roughness [30,31] is believed to be related to the crystallinity of the solid surface. Deconvolution of the XRD curves was used to verify that affirmation (Figure S3, Supplementary Material). The crystallinity of PHB microcapsules from 0.5% of PVA was 50.7% and is increased to 60.4% for those microcapsules from 2.0% PVA.

### 3.3. Encapsulation of Urea in Microcapsules

**Figure 2:**
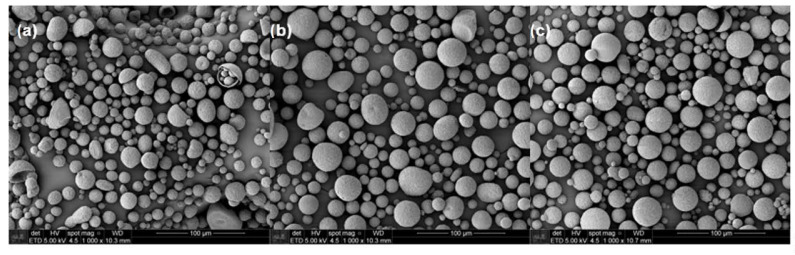
SEM micrographs of microcapsules with urea encapsulate of (**a**) polymer poly(3-hydroxybutyrate) (PHB), (**b**) PHB/MMT, and (**c**) PHB/OMMT.

Microcapsules having encapsulated urea were prepared using a continuous phase comprised of 0.5% PVA aqueous solution. The morphology and the microcapsules’ diameters were observed by SEM (Figure 2). The majority of the particles are characterized by spherical shapes; however, thanks to mechanical resistance given by the dispersed clay, microcapsules of PHB/MMT and PHB/OMMT presented fewer defects compared to those of PHB that have shown more elongated, collapsed and cracked particles.

**Figure 3:**
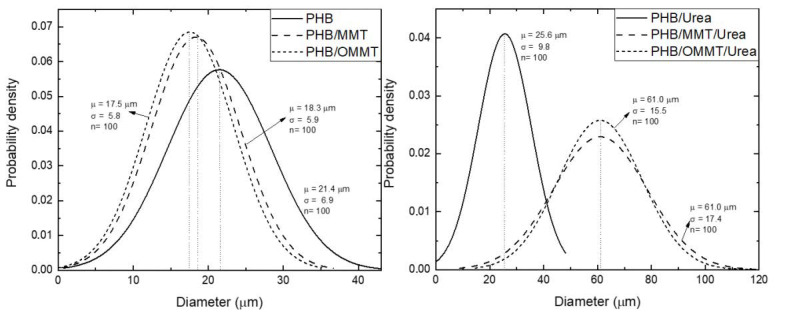
Normal distribution of diameter of PHB, PHB/MMT, and PHB/OMMT microcapsules with and without urea encapsulated.

The sizes of the microcapsules were also revealed by SEM, and the mean diameters and particle size distribution curves of all types of microcapsules were determined (Figure 3). All three types presented an increase in their mean diameter after the encapsulation of urea, although PHB ones present the smallest increase. In general, this type of particle had an increase of 20% in their diameter, while PHB/MMT and PHB/OMMT ones had an increase of 233% and 248%, respectively.

**Figure 4:**
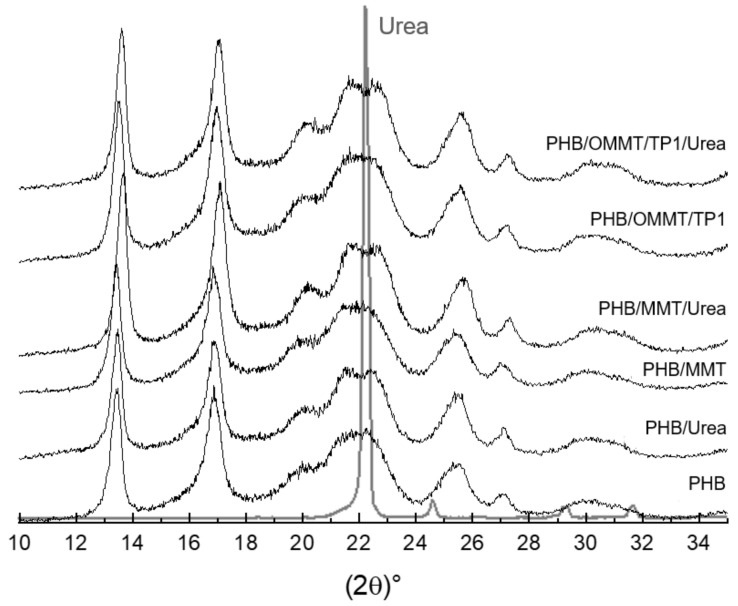
XRD patterns of microcapsules empty and with urea and urea.

XRD curves (Figure 4) were analyzed to identify the components in the nanocomposites. As shown in Figure 1, the basal peak of MMT and OMMT, around 6° and 4.9° (2ϴ), respectively, as well as that of kaolin, around 12°, are not observable in the XRD curves of the microcapsules. It suggests that the clays were well dispersed in the PHB matrix. To compare the influence of encapsulation, XRD curves of microcapsules, empty or with urea, were analyzed as well. The shape of peaks is quite similar among them; however, it is possible to observe that around 2ϴ = 22°, the peak of microcapsules with urea is more distinguishable than in empty microcapsules. This peak is the main peak of urea in its XRD curve and can be assumed as an indication that encapsulation was effective.

Microcapsule thermal stability was analyzed by TGA (Table 1). The microcapsules with encapsulated urea, independent of the polymer system, showed a minor shift to lower decomposition temperature compared to the corresponding empty one. Besides that, it can be observed that the PHB undergoes degradation without leaving residues; thus, the residue left by the nanocomposite microcapsules is due to the presence of clay. Residues in TGA analysis of polymer systems having dispersed heat resistant particles are also comprised of the char formed by the polymer itself as well as the polymer at the interface bound to the filler. Thus, the observed amount can surpass the inorganic component fraction and differences in quantities are indicative of the dissimilarities in the physical and chemical bonds between PHB and MMT or OMMT, allowing distinct organizations in the jellified aggregate state to form in the presence of PVA.

### 3.4. Microcapsules’ Biodegradation

**Figure 5:**
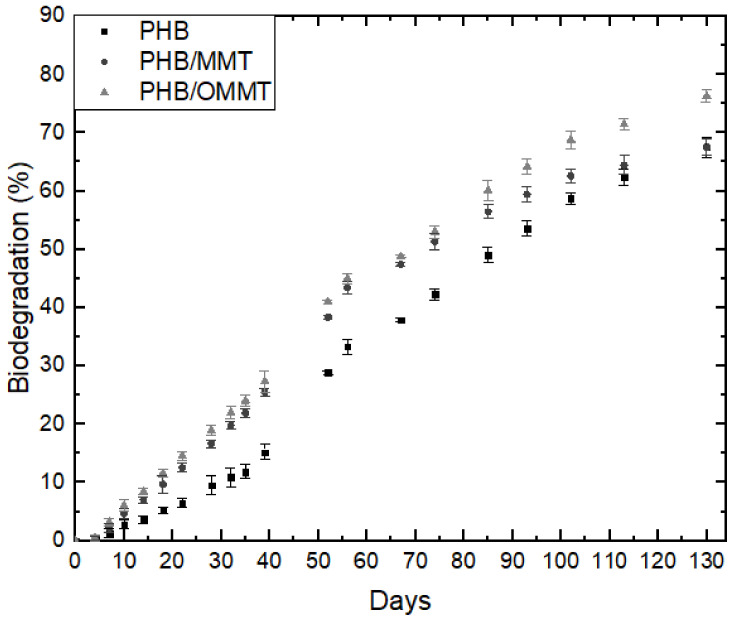
Biodegradation rates of microcapsules of PHB, PHB/MMT, and PHB/OMMT with urea encapsulated.

The biodegradation of microcapsules was carried out in sandy soil with a composition of approximately 71% of sand, 21% of silt, and 8% of clay, at pH 6, hence an acid soil. The maximum holding capacity of this soil was 44% (w), and the organic material was 16%. The rate of biodegradation can be observed in Figure 5, and nanocomposites microcapsules presented a higher degradation rate than PHB ones, which could be associated with the clay addition as it has hydroxyl groups in its surface, securing humidity and interacting with the hydrophobic PHB, contributing to initial microorganism colonization. In 130 days, approximately 75% of PHB/OMMT microcapsules have biodegraded, while around 65% of PHB and PHB/MMT. This occurred due to the fact that PHB/OMMT, although organophilized, was prepared using Tapioca Pure (TP), comprised of starchy carbohydrates, as a compatibilizer. Around the 100º day of degradation, there is a change in biodegradation rate, which goes to 5.2%, 3.1%, and 4.8% per week for PHB, PHB/MMT, and PHB/OMMT microcapsules, respectively. Consequently, if their rate remains constant, those materials will suffer complete degradation in 163, 170, and 156 days of incubation, respectively.

## 4. Conclusions

XRD, FTIR, and TGA analyses showed that clay organophilization was successful, and the weight of quaternary salt in the organoclay was estimated to be around 20.9%. XRD of nanocomposite microcapsules did not present de clays’ basal peak, which can mean that the clays were well dispersed in the PHB matrix.

The emulsion method influences the crystallinity and roughness of microcapsules. Microcapsules prepared with a lower concentration of PVA (0.5%) presented smother surfaces and lower crystallinity than those prepared with 2.0% of the emulsifier agent, which are 10% more crystalline and 38% rougher.

Urea encapsulation was verified through the size of microcapsules, XRD curves and TGA analyses. The increase of the mean size of microcapsules, around 240% in nanocomposites microcapsules, and 20% in PHB microcapsules indicate that nanocomposite microcapsules are more efficient in urea encapsulation. In microcapsules XRD curves, the urea peak around 22° is observed, which was not observed in XRD of empty microcapsules. TGA analysis showed that after the encapsulation, the initial degradation temperature of all types of microcapsules reduced, indicating an influence of the urea presence. All the types of PHB/MMT microcapsules showed a higher temperature of degradation, indicating better thermal stability. The biodegradation test showed that the addition of OMMT and TP in PHB improves the biodegradability of microcapsules, and it is expected to degrade completely faster than PHB and PHB/MMT microcapsules. Biodegradability properties in the soil of PHB microcapsules are not harmed by clay or compatibilizer presence, and microcapsules are completely degraded in approximately five months.

Future research will be pursued to identify the toxicity of PHB nanocomposites microcapsules in plants, identify the efficiency of urea encapsulation and study the release of urea through microcapsules.

## Figures and Tables

**Table 1:** Thermogravimetric analysis (TG) results of microcapsules.

Microcapsules Samples	PHB	PHB/Urea	PHB/MMT	PHB/MMT/ Urea	PHB/OMMT	PHB/OMMT/ Urea
Set degradation temperature (°C)	237	235	250	242	232	222
Residue (%)	0	0	1.1	4.5	4.3	5.3

## Data Availability

Not applicable.

## Supplementary Materials

The following are available online at https://www.mdpi.com/2073-4360/13/5/722/s1, Figure S1: Thermogravimetric curves of MMT and OMMT samples, Table S1: FTIR OMMT and MMT clays, Figure S2: SEM micrograph of microcapsules of PHB made with a) 0.5% and b) 2.0% of PVA. AFM micrograph of microcapsules of PHB made with (**a**) 0.5 and (**b**) 2.0% of PVA, Figure S3: XRD pattern of PHB microcapsules made with 0.5% and 2.0%.
