# Supplementary material for: Biodegradable Nanocomposite Microcapsules for Controlled Release of Urea
Source: Polymers (Basel). 2021 Feb 26;13(5):722. doi: 10.3390/polym13050722 (PMC7956393; doi:10.3390/polym13050722)
Supplement: Supplementary file 1 [file polymers-13-00722-s001.pdf]

Supplementary Information

Biodegradable nanocomposite microcapsules for controlled release of urea

Jessica de Carvalho Arjona [1,*], Maria das Graças Silva-Valenzuela [1], Shu-Hui Wang [1] and Francisco Rolando Valenzuela-Diaz [1]

[1] Department of Metallurgical and Materials Engineering, Polytechnic School, University of Sao Paulo. Av. Prof. Mello Moraes 2463, Sao Paulo 05508-030, Brazil; jessica.arjona@usp.br

* Correspondence: jessica.arjona@usp.br; Av. Prof. Mello Moraes 2463, Sao Paulo 05508-030, Brazil.

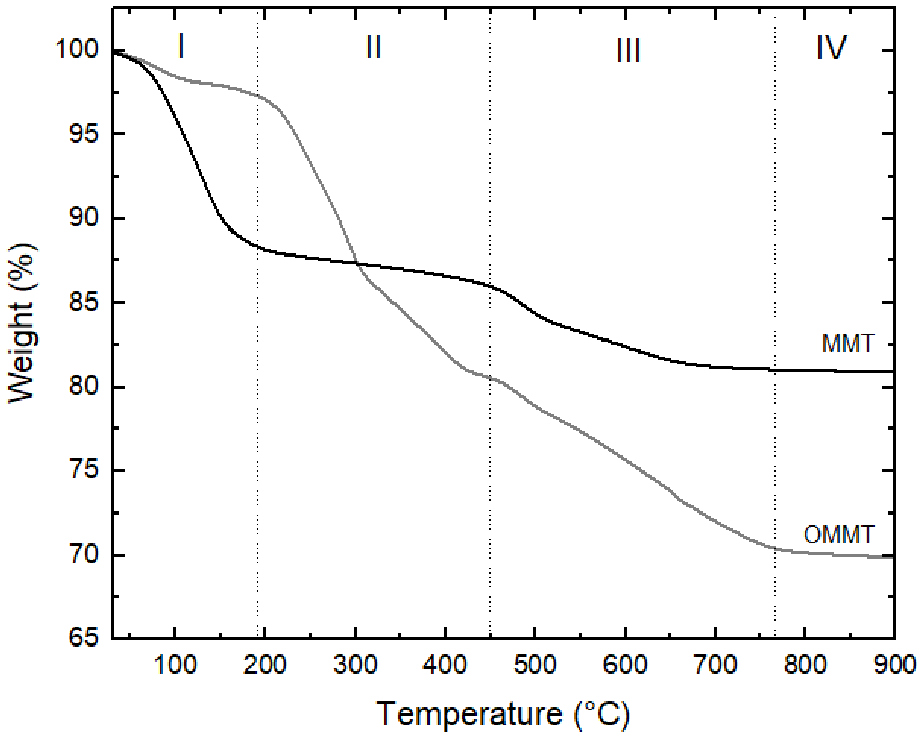

Figure S1. Thermogravimetric curves of MMT and OMMT samples.

Table S1. FTIR OMMT and MMT clays.

MMT	OMMT	Smectite	Kaolinite
693	693	Vibration of Si-O-Mg and Si-O-Mg bonds	-
815	815	Dioctahedral clays – Mg-Al-OH bending	-
915	915	Al-Al-OH deformation	OH deformation of inner hydroxyl groups
1,026	1,026	Stretching of bonds Si-O	-
1,108	1,108	Stretching of bonds Si-O	-
1,600	1,600	Vibration of O-H water molecules	OH deformation of water
-	2,838	scissor vibrations of C-H3 bonds	-
-	2,931	stretching of CH bonds	-
3,659	3,659	-	OH stretching of inner surface hydroxyl groups

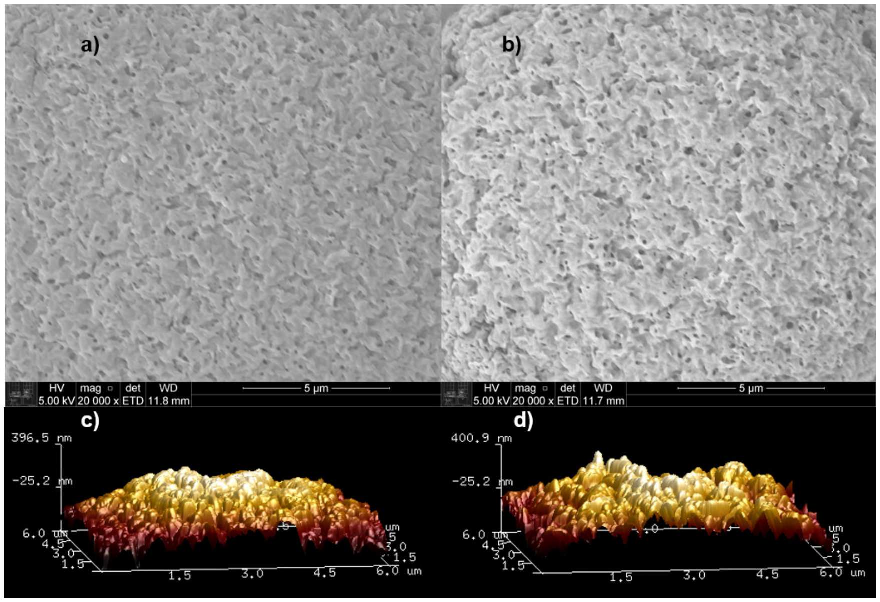

Figure S2. SEM micrograph of microcapsules of PHB made with a) 0.5% and b) 2.0% of PVA. AFM micrograph of microcapsules of PHB made with (**a**) 0.5 and (**b**) 2.0% of PVA.

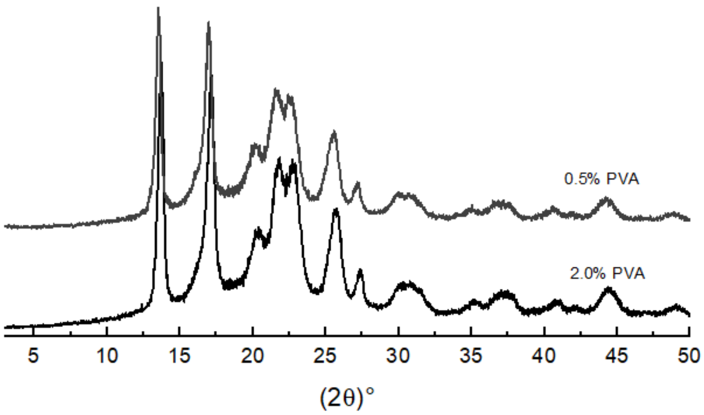

Figure S3. XRD pattern of PHB microcapsules made with 0.5% and 2.0%
